# Cornual abscess rupture: A rare etiology of acute abdomen

**DOI:** 10.1002/ccr3.1855

**Published:** 2018-10-18

**Authors:** Pooja Patil, Lannah L. Lua, Bobby Brar, Mary Froehlich, Petar Planinic

**Affiliations:** ^1^ Department of Obstetrics and Gynecology University of Nevada Las Vegas School of Medicine Las Vegas Nevada; ^2^ University of Nevada Reno School of Medicine Reno Nevada

**Keywords:** acute abdomen, cornual abscess, pelvic infection, peritonitis, pyometra, retained intrauterine device

## Abstract

Ruptured cornual abscess or pyometra can resemble other more common causes of acute abdomen, including appendicitis, diverticulitis, tubo‐ovarian abscess, and perforated viscus. Despite its rarity, the diagnosis of ruptured pyometra should always be considered in females presenting with acute abdominal pain, particularly in the setting of a retained intrauterine device.

Question: What is the diagnosis?

A 42‐year‐old female presented with acute abdominal pain and imaging findings shown in Figure 1. A retained intrauterine device (IUD) was removed at bedside. Intravenous antibiotics were initiated for presumed tubo‐ovarian abscess (TOA). On hospital day 3, she developed acute worsening of clinical and imaging findings (Figure 2).

**Figure 1:**
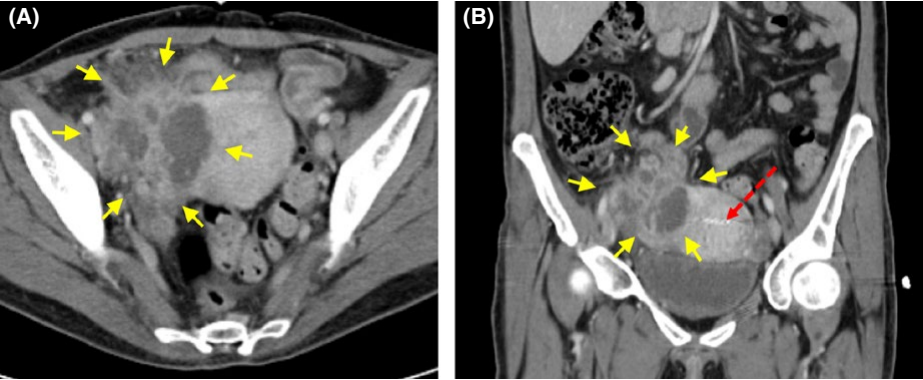
Computed tomography (CT) scan of the abdomen and pelvis of a female presenting with acute abdominal pain and found to have right lower quadrant tenderness with guarding, cervical motion tenderness, leukocytosis (white blood cell count of 15.9 × 10^9^ cells/L), and vital signs within normal limits. Imaging demonstrated heterogeneous solid and cystic soft tissue mass in the right adnexal region with surrounding enteric inflammatory changes, suggestive of appendiceal abscess vs tubo‐ovarian abscess. Overall area of involvement measures 9 × 7 × 6 cm (yellow arrows). A retained intrauterine device (red arrow) of 17 years was also visualized. A, Axial view. B, Coronal view

**Figure 2:**
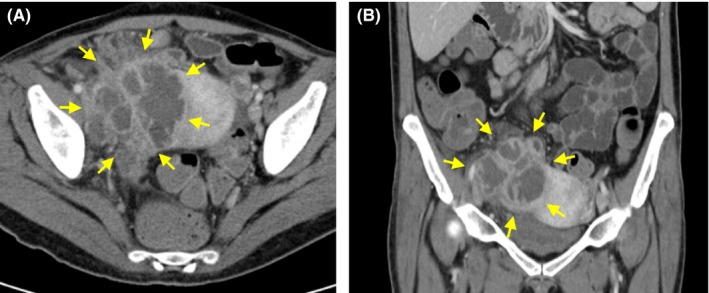
CT scan of abdomen and pelvis on hospital day 3 was obtained due to acute worsening of clinical picture, including development of generalized abdominal tenderness, persistent fevers up to 102° Fahrenheit, and worsening leukocytosis. Imaging findings demonstrated a septated soft tissue collection in the right adnexal region (yellow arrows) that has increased in size compared to imaging on admission. Again, this collection may represent an enlarging appendiceal abscess vs tubo‐ovarian abscess. A, Axial view. B, Coronal view

Answer: Exploratory laparotomy revealed a ruptured right cornual abscess (Figure 3).

**Figure 3:**
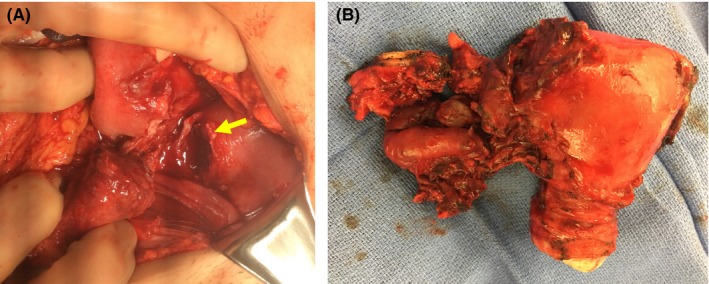
A, Intraoperative findings were notable for an 8‐cm uterus with a 3‐cm area of abscess rupture originating from the right cornu of the uterus, leaving a large uterine defect (yellow arrow). Spillage of pus into the abdominal cavity was noted immediately upon entry, as well as exudates and adhesions involving the cecum, appendix, small bowel, and pelvic structures. B, Hysterectomy with right salpingo‐oophorectomy was performed, and the specimen was sent to pathology. The appendix and right adnexa were markedly inflamed but were not the primary source of acute infection. The patient improved clinically following hysterectomy with salpingo‐oophorectomy and was discharged on hospital day 6

Pyometra, a uterine infection, generally occurs in postmenopausal women in the setting of gynecologic malignancy.^1^ Its incidence is 0.03%‐0.11%.^1^ Malignancy‐associated pyometra develops from cervical occlusion/stenosis and radiation cervicitis.^1^ Long‐term IUD, another risk factor, accounts for 28.6% of cases. Pyometra rupture with IUD is explained by age‐related uterine involution, circulatory insufficiency, and decreased immunity.^2^ This case highlights the importance of counseling patients regarding timely IUD removal and risks with long‐term use.

Diffuse peritonitis resulting from ruptured pyometra can be observed in other gynecologic (TOA, endometritis, pelvic inflammatory disease, adnexal torsion) and non‐gynecologic (appendicitis, diverticulitis, cholecystitis, pancreatitis, bowel obstruction/ischemia, perforated viscus) conditions. Imaging can help delineate the source; however, in this case, imaging was equivocal for TOA vs appendicitis, making definitive diagnosis difficult. Prompt diagnosis of pyometra is critical, as mortality approaches 25% with rupture.^1^ In an unruptured uterus, mainstay of treatment is dilation, abscess drainage, curettage, and intravenous antibiotics.^1^ With rupture, emergent surgical intervention is warranted with peritoneal lavage and hysterectomy with salpingo‐oophorectomy.^2^ Management also becomes increasingly complex, as septic shock may ensue with abscess rupture, necessitating intensive care and prolonged hospitalization. Infection can recur/persist in up to 33% of cases^1^; therefore, close surveillance following discharge is crucial.

## CONFLICT OF INTEREST

None declared.

## AUTHOR CONTRIBUTIONS

LL/PP: participated in the care of the patient, manuscript writing and editing, and literature review. BB: participated in manuscript writing and editing. MF: participated in the care of the patient and literature review. PP: participated in the care of the patient and supervised manuscript preparation.

## References

[1] Bacanakgil BH , Yıldırım SG . Postmenopausal pyometra related to forgotten intrauterine device. Radiol. Infect. Dis. 2018 10.1016/j.jrid.2018.01.001.

[2] Li CH , Chang WC . Spontaneous perforated pyometra with an intrauterine device in menopause: a case report. Jpn. J. Infect. Dis. 2008;61:477‐478. 19050359

